# Rapid molecular assays for the detection of the four dengue viruses in infected mosquitoes

**DOI:** 10.12688/gatesopenres.13534.2

**Published:** 2022-12-22

**Authors:** Madeeha Ahmed, Nina M. Pollak, Leon E. Hugo, Andrew F. van den Hurk, Jody Hobson-Peters, Joanne Macdonald

**Affiliations:** 1 Centre for Bioinnovation, University of the Sunshine Coast, Sippy Downs, QLD, 4556, Australia; 2 School of Science, Technology and Engineering, University of the Sunshine Coast, Sippy Downs, QLD, 4556, Australia; 3 DMTC Limited, Hawthorn, Victoria, 3122, Australia; 4 Mosquito Control Laboratory, QIMR Berghofer Medical Research Institute, Herston, QLD, 4006, Australia; 5 Public Health Virology, Forensic and Scientific Services, Department of Health, Queensland Government, Coopers Plains, QLD, 4108, Australia; 6 Australian Infectious Diseases Research Centre, School of Chemistry and Molecular Biosciences, The University of Queensland, St Lucia, QLD, 4072, Australia

**Keywords:** dengue virus, recombinase polymerase amplification, isothermal amplification, lateral flow detection, rapid molecular assays, mosquitoes, Aedes aegypti, mosquito surveillance

## Abstract

The pantropic emergence of severe dengue disease can partly be attributed to the co-circulation of different dengue viruses (DENVs) in the same geographical location. Effective monitoring for circulation of each of the four DENVs is critical to inform disease mitigation strategies. In low resource settings, this can be effectively achieved by utilizing inexpensive, rapid, sensitive and specific assays to detect viruses in mosquito populations. In this study, we developed four rapid DENV tests with direct applicability for low-resource virus surveillance in mosquitoes. The test protocols utilize a novel sample preparation step, a single-temperature isothermal amplification, and a simple lateral flow detection. Analytical sensitivity testing demonstrated tests could detect down to 1,000 copies/µL of virus-specific DENV RNA, and analytical specificity testing indicated tests were highly specific for their respective virus, and did not detect closely related flaviviruses. All four DENV tests showed excellent diagnostic specificity and sensitivity when used for detection of both individually infected mosquitoes and infected mosquitoes in pools of uninfected mosquitoes. With individually infected mosquitoes, the rapid DENV-1, -2 and -3 tests showed 100% diagnostic sensitivity (95% CI = 69% to 100%, n=8 for DENV-1; n=10 for DENV 2,3) and the DENV-4 test showed 92% diagnostic sensitivity (CI: 62% to 100%, n=12) along with 100% diagnostic specificity (CI: 48–100%) for all four tests. Testing infected mosquito pools, the rapid DENV-2, -3 and -4 tests showed 100% diagnostic sensitivity (95% CI = 69% to 100%, n=10) and the DENV-1 test showed 90% diagnostic sensitivity (55.50% to 99.75%, n=10) together with 100% diagnostic specificity (CI: 48–100%). Our tests reduce the operational time required to perform mosquito infection status surveillance testing from > two hours to only 35 minutes, and have potential to improve accessibility of mosquito screening, improving monitoring and control strategies in low-income countries most affected by dengue outbreaks.

## Introduction

Dengue is a debilitating and potentially fatal mosquito-borne disease with increasing global incidence over the last three to four decades ^1^. Dengue occurs in approximately 129 countries ^2^, putting four billion people at risk of severe disease, with an estimated 390 million people infected each year ^3^. The disease is caused by infection with dengue viruses (DENVs), which exist as four antigenically and genetically distinct serotypes (DENV 1–4) ^4^. Subsequent infections with different serotypes can present as potentially fatal severe dengue, due to antibody-dependent enhancement ^3, 5^. The pantropic emergence of severe dengue disease can be partly attributed to the co-circulation of different serotypes in the same geographical location ^6^.

The primary vectors that transmit DENVs are the urbanized mosquitoes, *Aedes aegypti* and *Aedes albopictus* ^7^. Surveillance of different DENV serotypes in these vectors is critical for monitoring transmission dynamics and risk of cross-serotype secondary infections ^8^. Mosquito-based surveillance also plays an important role in identifying active transmission that could lead to outbreaks, designing effective mitigation programs and assessing the effectiveness of implemented control strategies ^9^. Methods for DENV surveillance in mosquitoes include virus isolation ^10– 12^, antigen detection ^13^, and molecular testing using either conventional ^14– 17^ or reverse-transcription quantitative polymerase chain reaction (RT-qPCR) ^18– 20^ or isothermal amplification techniques ^21– 25^.Apart from isothermal amplification, these techniques can be limited in their implementation due to issues with sensitivity or specificity, or because they require sophisticated instruments and laboratory infrastructure that is not readily available in resource-limited countries where DENVs exert their greatest toll.

For rapid detection of DENV in resource-limited environments, the isothermal technique recombinase polymerase amplification (RPA) has been identified as a promising tool, with rapid reaction times (15–30 minutes) at a single constant low temperature, without the need for sophisticated instruments ^25^. The technology has demonstrated diagnostic sensitivity and specificity comparable to RT-qPCR (77–98% clinical sensitivity, n=31, 97.9–100% clinical specificity) ^26– 30^ when used to detect DENVs in a fluorescent real-time reverse-transcription format (real-time RT-RPA), as well with reverse-transcription RPA lateral flow detection (RT-RPA/LFD), which showed a 100% coincidence rate with RPA and RT-qPCR using clinical samples ^31^. However, these RPA-based tests required multi-step column-based kits to extract viral nucleic acids that required laboratory-based testing, did not distinguish between different serotypes, and were not tested for utility with mosquito surveillance. In this study, we report four rapid RT-RPA/LFD tests able to specifically detect each of the four DENVs. These tests were then combined with a novel 10-minute field-amenable procedure for mosquito sample processing preparing total nucleic acids that only requires a single-tube and two simple steps and does not need any sophisticated instruments or specialized viral RNA extraction kits. The resultant rapid DENV 1–4 tests were then tested using experimentally infected mosquitoes. Our rapid dengue 1–4 tests have potential to provide low-resource implementation of specific DENV detection in mosquito populations enabling effective surveillance for improved disease management.

## Methods

### Oligonucleotides, plasmids, and RNA standards

***Plasmids*** containing synthetic non-structural five (NS5) gene fragments from the respective dengue serotypes in pBIC-A cloning vector were obtained from Bioneer Pacific (Victoria, Australia) to use as a control DNA template. Synthetic NS5 gene fragments in pBIC-A plasmids were derived from isolates of each dengue serotype: DENV-1 (GU131962.1) from 885 to 1561 bp (677 bp); DENV-2 (NC_001474.2) from 874 to 1560 (687 bp); DENV-3 (KF921921.1) from 865 to 1562 (698 bp) and DENV-4 (JF262780.1) from 864 to 1559 bp (696 bp) as a template. A fifth plasmid containing synthetic DENV-1 capsid fragment conjoined to the NS5 fragment was constructed to be used as a molecular standard for a universal dengue RT-qPCR test. This plasmid was also obtained from Bioneer Pacific (Victoria, Australia), and covered 200 bp of the anchored capsid peptide coding region (95 to 436 nt of Genbank accession number: KF973466.1) and 230 bp of the NS5 gene fragment (900 to 1130 nt of Genbank accession number: GU131962.1) in pBIC-A cloning vector. Copy number was determined using the Qubit™ DNA HS Assay Kit (Thermo Fisher Scientific Australia Pty Ltd, Victoria, Australia) according to the manufacturer’s instructions.

****RNA transcripts**** for sensitivity studies were derived from the respective plasmid DNA. Briefly, the circular plasmid was linearized with Xho-1 restriction enzyme to obtain the template, followed by agarose gel electrophoresis, extraction and gel clean-up of the digested linear template DNA (NucleoSpin® Gel and PCR Clean-up: Macherey-Nagel, Düren, Germany). Single-stranded RNA was then synthesized by *in vitro* transcription from the linear DNA template using the MEGAscript® T7 transcription kit (Invitrogen by Thermo Fisher Scientific Australia Pty Ltd, Victoria, Australia). Transcribed RNA was quantified using the Qubit™ RNA HS Assay Kit (Thermo Fisher Scientific Australia Pty Ltd, Victoria, Australia) according to the manufacturer’s protocol for the calculation of copy numbers and stored at -80°C.

****Oligonucleotide primers and probes**** for the dengue virus serotype-specific RPA test were designed from the consensus sequence of the NS5 gene coding region, which is conserved across several species of flaviviruses including dengue viruses ^32^. The design of the consensus oligos involved the sequences of all the genotypes representing each dengue serotype, according to criteria described by the manufacturer (TwistDX). A total of 1,703, 1,313, 930 and 184 published NS5 gene sequences were aligned for DENV 1, 2, 3 and 4, respectively, to identify conserved regions in the target sequences for primer design. Primers and probes were synthesized by Bioneer Pacific (Victoria, Australia) using HPLC and PAGE purification, respectively. At least five different forward primers, three reverse primers, and two probes were designed for each of the DENV assays and tested in various combinations using standard plasmid DNA and synthetic RNA transcripts to optimize the respective serotype-specific DENV RPA-LFD assays, which use selected primer and probe sequences ( Table 1). Primers and probes for the universal dengue RT-qPCR test were synthesized by Integrated DNA technologies (Iowa, USA) using standard desalting and HPLC purification, respectively.

**Table 1.** Selected primer and probe sequences for the Dengue 1-4 RT-RPA/LFD test.

RAPID TEST	NAME	SEQUENCE
**DENV-1**	D1-F3	CATGGATCATATGAGGTCAARCCATCAGGATCAGC
	D1-R3	[5' Biotin] GTCACCTCCATRATTTGTGCTGTGCCTCGTTTYGC
	D1-P2	[5' FAM] CATGGTCACACAAATAGCYATGACTGAYAC [Internal dS Spacer] ACACCCTTYGGACAAC [3' C3 spacer]
**DENV-2**	D2-F3	GGNAGCTANGAAACAAAACANACTGGATCAGCA
	D2-R2	[5' FAM] GGGTTCTCGTGTCCACTTTYTCTTTRAAAACRCGC
	D2-P1	[5' Biotin] GRYTGCTRACMAAACCTHGGGAYGTYVTYCC [Internal dS spacer] AYGGTRACACARATGG [3' C3 spacer]
**DENV-3**	D3-F5	CTTACCAYGGATCBTATGAAGTHAARGCCACAGGC
	D3-R1	[5' FAM] GTGTCCACTTTCTCTTTRAARACYCTYTGCTGSCC
	D3-P1	[5' Biotin] GATAAAYGGAGTYGTGAAACTYCTCACNAA [Internal dS spacer] CCRTGGGATGTGGTKCC [3' C3 spacer]
**DENV-4**	D4-F2	GAAGCTATGAAGCYCCYTCGACAGGCTCDGCNTCYTC
	D4-R1	[5' FAM] GTRTCNACCTTCTCYTTRAACACTCTYTGTTGCCC
	D4-P1	[5' Biotin] GGGAYGTRRTTCCRATGGTGACYCAGTTRGC [Internal dS spacer] ATGACAGAYACAACCC [3' C3 spacer]

### Viruses and mosquitoes

**Virus strains.** The four DENVs that were used to infect mosquitoes were from stocks produced from previously described virus strains (DENV-1 ET00.243, JN415499; DENV-2 ET00.300, JN568254; DENV-3 East Timor 2000, JN575566; DENV-4 ET00.288, JN575585) ^33^. Other flavivirus strains originally derived from clinical isolates included Zika virus (ZIKV MR766), Japanese encephalitis virus (JEV Nakayama strain), Murray Valley encephalitis virus (MVEV 1–51 strain) and West Nile virus Kunjin strain (WNV _KUNV_ NSW2011 strain). **Cell culture.** *Aedes albopictus* clone C6/36 cells (ATCC® CRL-1660™) were cultured in RPMI 1640 medium (Thermo Fisher Scientific Australia Pty Ltd, Victoria, Australia) supplemented with L-glutamine (2 mmol/L; Gibco by Thermo Fisher Scientific Australia Pty Ltd, Victoria, Australia), 5% heat-inactivated fetal bovine serum (FBS; Sigma-Aldrich, New South Wales, Australia), penicillin (50 units/mL) and streptomycin (50 µg/mL) at 28°C and 5% CO _2_. Prior to reaching confluency, the cells were treated with 0.25% trypsin solution (Gibco by Thermo Fisher Scientific Australia Pty Ltd, Victoria, Australia) and resuspended in fresh growth medium and seeded on new growth surfaces.

**Virus culture.** DENV 1–4 strains and the other flavivirus strains were propagated to a concentration of 10 ^5^ to 10 ^7^ tissue culture infectious dose (TCID) _50_/mL in T25 culture flasks seeded with C6/36 cells in RPMI 1640 growth media as described above, with the exception that 2% FBS was used. Seven days post infection, 2 mL of TRI Reagent (Sigma-Aldrich, New South Wales, Australia) was added to the flask preparation and swirled over the cell area for one to two minutes. To prepare the inactivated viruses for extraction the inoculum was separated into a tube and centrifuged at 3000 rpm for 10 minutes at 4°C to separate supernatant from cell pellet. The total RNA was extracted from the infected culture cell lysate stocks using the TRI Reagent extraction according to manufacturer’s instructions, resuspended in nuclease-free water, quantified using a NanoDrop 2000 spectrophotometer (Thermo Fisher Scientific Australia Pty Ltd, Victoria, Australia) and stored at -80°C.

**Exposure of mosquitoes to DENVs.** *Aedes aegypti* (Innisfail strain) were inoculated with 200 nL of 10 ^4^–10 ^5^ TCID _50_/mL of DENV 1, 3 and 4 via intrathoracic microinjection. Mosquitoes were exposed to DENV-2 via feeding on an infectious blood meal containing 10 ^7^ TCID _50_/mL of virus, as part of previously reported experiments ^34^. Mosquitoes not exposed to virus were used as uninfected controls. At eight days post-exposure, mosquitoes were anaesthetised with CO _2_ gas before being stored at -80°C. Mosquito heads were severed and used for RT-qPCR testing to assess infection status, whereas the bodies were kept for rapid DENV 1–4 testing.

### Mosquito sample RNA extraction and RT-qPCR testing

Each of the uninfected or infected *Aedes aegypti* mosquitoes had the head separated from the body using a sterilized scalpel. Then, individual or pooled mosquito heads were manually homogenized in 150 µL phosphate-buffered saline (PBS) using polypropylene pestles as described previously by Li *et al.*, ^21^. Total RNA was extracted using the QIAmp Viral RNA Mini Kit (Qiagen, Hilden, Germany) according to the manufacturer’s instructions, performing a double elution using 2 x 40 µL buffer AVE supplied in the kit. Extracts were tested using universal DENV 1–4 reverse-transcription quantitative PCR assay (RT-qPCR DU5 MGB2017) designed to target the capsid peptide coding region as previously described by Pyke *et al.* ^35^ using the SuperScript™ III Platinum™ One-Step qRT-PCR Kit (Thermo Fisher Scientific Australia Pty Ltd, Victoria, Australia). Kit-extracted mosquito head samples were amplified in a 20 µL reaction containing reaction mix (1X), ROX reference dye (50 nM), SuperScript ^TM^ III/Platinum ^TM^ Taq Mix (1X), two forward primers (900 nM each, DU5-F1: GAAYAACCAACGRAARAAGRCG; DU5-F2: ATGAACCAACGRAARAAGGTGG) and three reverse primers (300 nM each, DU5-R13: GAGAATCTCTTCGCCAACTGTG; DU5-R2: TGAGAATCTCTTYGTCARCTGYTG; DU5-R4: GAGAATCTCTTCACCAACCCTTG), MGB probe (150 nM, DU5-MGB2017: 6FAM – AATATGCTGAAACGCG - MGBNFQ ), nuclease free water, and 5 uL sample. An *in vitro* transcribed RNA standard (DENV-1 cap-NS5) was used for absolute quantification by RT-qPCR. Amplification was performed using the Rotorgene Q real-time thermocycler (Qiagen, Hilden, Germany). The thermal profile consisted of a five-minute RT step at 50°C, two-minute *Taq* polymerase activation step at 95°C followed by 40 cycles of PCR at 95°C for 15 seconds (denaturation) and 60°C for one minute (annealing and extension).

### Rapid DENV (1–4) tests

***Sample processing.*** Individual mosquito bodies were homogenized with a pestle in 50 µL of Sample Preparation Reagent (TNA-Cifer Reagent; BioCifer, Buderim, Australia) and incubated on ice for 10 minutes. Extracts were then immediately diluted 1:5 in RNase-free water, and 1 µL of this extract was used as a template for the subsequent RT-RPA reaction. For testing mosquito pools containing five mosquitoes, 125 µL Sample Preparation Reagent was used for homogenization, followed by 1:10 dilution in RNase-free water, to reduce the amount of potential inhibitory by-products in the mosquito homogenate ^36^.

****DENV reverse-transcription RPA tests (DENV 1–4 RT-RPA) for mosquito and RNA transcript testing.**** Each respective DENV 1–4 test was prepared separately using the TwistAmp™ nfo kit (TwistDX, Cambridge, United Kingdom), with final reaction conditions of 1x rehydration buffer and 1/5 rehydrated lyophilized pellet, forward primer (420 nM), reverse primer (420 nM), and probe (120 nM). For homogenized mosquito testing, Ribolock (10 U) and Moloney Murine Leukemia virus reverse transcriptase (mMLV, 40 U) were included with the individual DENV 1–4 RPA tests, along with 1 µL extracted RNA and magnesium acetate (14 mM), to a final reaction volume of 10 µL. For testing RNA-transcripts, betaine (120 mM) was also included with the DENV 1–4 RT-RPA tests. Reactions were incubated at 39°C for 20 minutes before lateral flow detection.

For plasmid DNA control testing, Ribolock, mMLV, and betaine were excluded. 1 µL DNA sample was added before activation by magnesium acetate (14 mM), to a final reaction volume of 10 µL. Reactions were incubated for 15 minutes at 39°C before lateral flow detection.

****Lateral flow detection (LFD).**** After the RPA (DNA plasmid control) or RT-RPA (RNA transcripts and mosquitoes) incubation, 2 µL of the amplified RPA reaction mix was added to the sample pad of the lateral flow strip (Milenia Biotec, Giessen, Germany), which had been pre-activated by the addition of 8 µL 0.4% casein in PBST to the sample pad ^37^. Strips were then placed in 100 µL running buffer (100 mM H _3_BO _3_, 100 mM Na _2_B _4_O _7_, 1% bovine serum albumin, 0.05% Tween 20, pH 8.8) ^38^ for five minutes at room temperature, analyzed visually and photographed using a digital camera (MultiDoc-ItTM Digital Imaging System: Upland, CA, USA) or scanned using the Epson Perfection V39 Flatbed Scanner (Epson, New South Wales, Australia). On visual analysis, a single control line depicted the absence of DENV and the appearance of two lines *i.e*., a test line along with the control line indicated the presence of DENV.

****Image analysis.**** Greyscale converted images were analyzed using ImageJ software (bundled with 64-bit 1.8.0_172, National Institute of Health, USA; RRID:SCR_003070) ^39^ by measuring the mean grey value and subtracting it from the maximum grey value for a given area. The average of two neighbouring white spaces is subtracted from the band intensity to normalize the results for each test band. A sample was classified as positive when the normalized band intensity was three times higher than the standard deviation of the two neighbouring white space values ^40^.

## Results

### Analytical sensitivity

**Figure 1.**
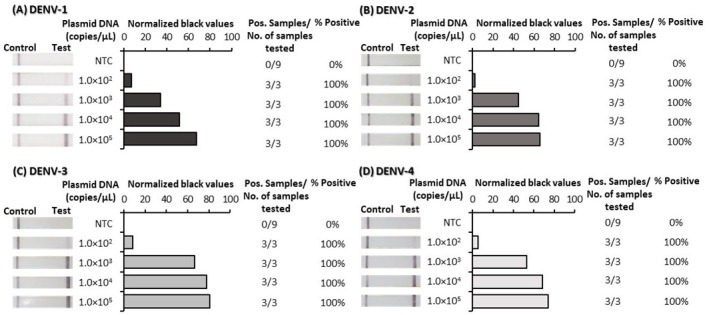
Analytical sensitivity determination of dengue virus (DENV) 1–4 recombinase polymerase amplification lateral flow detection (RPA-LFD) tests using plasmid DNA. Sensitivity testing used non-structural 5 (NS5) gene fragment plasmid DNA diluted 10-fold in water for DENV-1 RPA-LFD ( **A**), DENV-2 RPA-LFD ( **B**), DENV-3 RPA-LFD ( **C**) and DENV-4 RPA-LFD ( **D**). Images of lateral flow strips (LFS) with two bands (control and test band) indicates the sample is positive for respective DENV plasmid DNA, and single control band indicates a valid reaction with negative sample. Photograph of lateral flow strips with control bands (all samples) and test bands (positive samples) compared to copy number of serially diluted plasmid DNA (copies/μL) and no template control (NTC) (left). Normalised pixel density (normalised black values) from the lateral flow test strip displayed (middle). Positive (Pos.) results compared to number (No.) of samples tested at that dilution was used to calculate the percentage of positive tests performed at that dilution (right).

**Figure 2.**
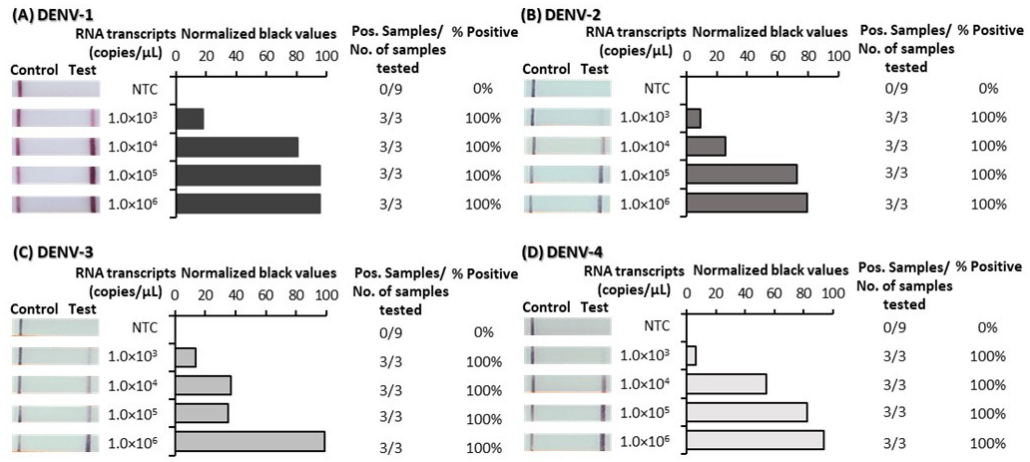
Analytical sensitivity determination of dengue virus (DENV) 1–4 reverse-transcription recombinase polymerase amplification lateral flow detection (RT-RPA/LFD) tests using *in vitro* transcribed RNA. Sensitivity testing used non-structural 5 (NS5) gene fragment RNA transcripts diluted 10-fold in water for DENV-1 RT-RPA/LFD ( **A**), DENV-2 RT-RPA/LFD ( **B**), DENV-3 RT-RPA/LFD ( **C**) and DENV-4 RT-RPA/LFD ( **D**). Images of lateral flow strips (LFS) with two bands (control and test band) indicates the sample is positive for respective DENV RNA transcript, and single control band indicates a valid reaction with negative sample. Photograph of lateral flow strips with control bands (all samples) and test bands (positive samples) compared to copy number of serially diluted template RNA (copies/μL) and no template control (NTC) (left). Normalised pixel density (normalised black values) from the lateral flow test strip displayed (middle). Positive (Pos.) samples compared to number (No.) of samples tested at that dilution was used to calculate the percentage of positive tests performed at that dilution (right).

To develop rapid, low-resource, and serotype-specific DENV tests, for each DENV serotype, we designed RPA tests that targeted a conserved region within the dengue NS5 gene. Primers and probes were designed to enable subsequent lateral flow detection (LFD) ^37, 40, 41^, such that appearance of a test line signified positive detection of each of the DENVs. RPA primer and probe optimization was performed first using plasmid DNA and was subsequently confirmed to be suitable for reverse-transcription RPA (RT-RPA) using RNA transcripts. The analytical sensitivity of the final optimized tests was determined using 10-fold serial dilutions of both plasmid DNA ( Figure 1) and RNA-transcripts ( Figure 2); underlying data ^42^. Test lines were observed by eye to appear for very low concentrations of both plasmid DNA and RNA transcripts. ImageJ analysis of test band intensity revealed the DENV 1–4 RPA/LFD tests detected down to 100 copies/µL of plasmid DNA ( Figures 1A–D) and the DENV 1–4 RT-RPA/LFD tests consistently detected down to 1,000 copies/µL of RNA transcripts ( Figures 2A–D).

### Analytical specificity

**Figure 3.**
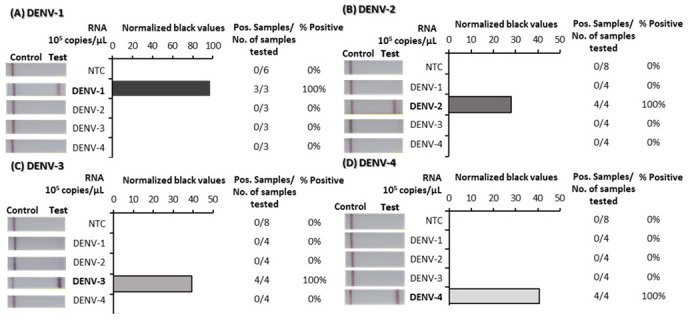
Analytical specificity trials of dengue virus (DENV) 1–4 reverse-transcription recombinase polymerase amplification lateral flow detection (RT-RPA/LFD) tests using *in vitro* transcribed RNA. Non-structural 5 (NS5) gene fragment RNA transcripts specific to each DENV serotype were tested for detection by each specific RT-RPA/LFD test: DENV-1 ( **A**), DENV-2 ( **B**), DENV-3 ( **C**) and DENV-4 ( **D**). Images of lateral flow strips (LFS) with two bands (control and test band) indicates the sample is positive for respective DENV RNA, and single control band indicates a valid reaction with negative sample (left). Normalised pixel density (black values) from the lateral flow test strip test bands of each DENV template RNA (10 ^5^ copies/μL) and no template control (NTC) displayed (middle). Positive (Pos.) samples compared to number (No.) of samples tested were used to calculate percentage of positive samples (right).

**Figure 4.**
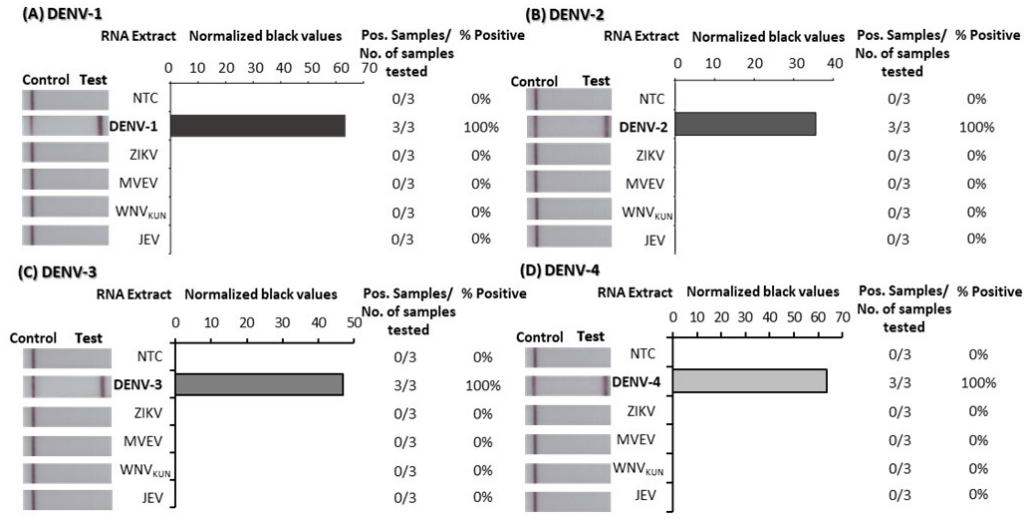
Analytical specificity testing of dengue virus (DENV) 1–4 reverse-transcription recombinase polymerase amplification lateral flow detection (RT-RPA/LFD) tests against other flavivirus RNA extracts. Specificity was tested using viral RNA extracts from the flaviviruses Zika virus (ZIKV), Murray Valley encephalitis virus (MVEV), West Nile virus Kunjin strain (WNV _KUN_) and Japanese encephalitis virus (JEV) for DENV-1 ( **A**), DENV-2 ( **B**), DENV-3 ( **C**) and DENV-4 ( **D**) RT-RPA/LFD assays. Images of lateral flow strips (LFS) with two bands (control and test band) indicates the sample is positive for respective DENV synthetic RNA transcript, and single control band indicates a valid reaction with negative sample. Nuclease-free water was tested as the no template control (NTC; left). Normalised pixel density (black values) from the assay displayed (middle). Positive (Pos.) samples compared to number (No.) of samples tested using different viral RNA extracts were used to calculate percentage of positive samples (right).

To confirm the specificity of the DENV 1–4 RT-RPA/LFD assays, tests were performed using high concentrations (10 ^5^ copies/µL) of RNA transcripts from each of the four DENVs. For each assay, a strong test band indicated that each test was specific to its virus specific target and did not detect any of the other three DENVs ( Figure 3; underlying data ^42^). We also confirmed the DENV-specific tests could not detect other flaviviruses by trialling detection of RNA extracted from ZIKV, JEV, MVEV, and WNV _KUN_ cell culture supernatant (5–7 log _10_ TCID _50_/ml) ( Figure 4; underlying data ^42^). No test lines appeared when any of the respective flaviviral RNA-extracts were applied to any of the DENV 1–4 RT-RPA/LFD tests, indicating that the assays were 100% specific to the respective DENVs they were designed to detect.

### Detection of dengue-infected mosquitoes

**Figure 5.**
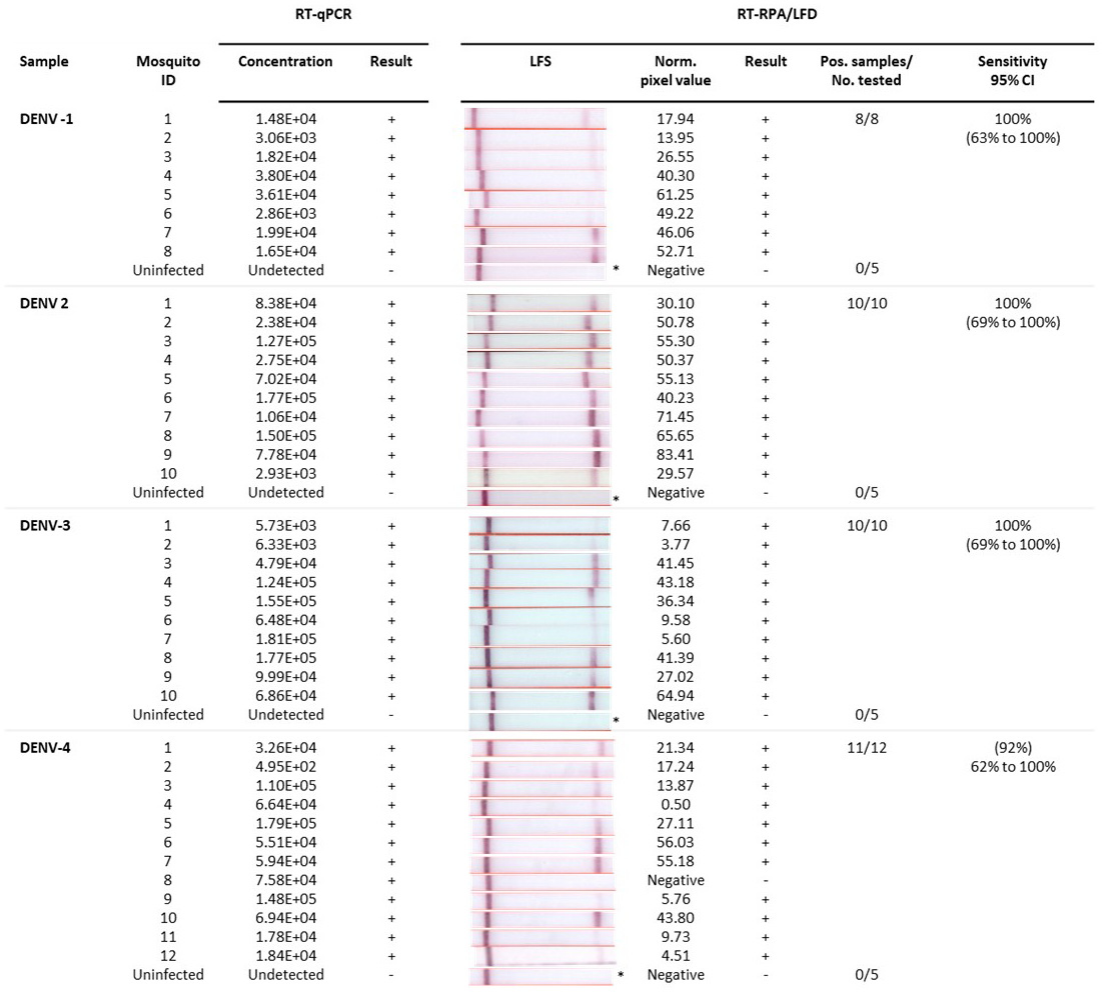
Detection of uninfected and infected individual mosquitoes by rapid dengue virus (DENV) 1–4 reverse-transcription recombinase polymerase amplification lateral flow detection (RT-RPA/LFD) assays and reverse-transcription quantitative polymerase chain reaction (RT-qPCR). Sample: Individual mosquitoes infected with DENV-1, DENV-2, DENV-3, DENV-4; Mosquito ID: Serotype-specific mosquito identification (ID) number; RT-qPCR: concentration (copies/µL) of each mosquito head extract tested and corresponding result (+/-); RT-RPA/LFD: Image of lateral flow strip (LFS) result from RT-RPA/LFD testing the corresponding mosquito body extract using the respective serotype RT-RPA/LFD test [Images of lateral flow strips with two bands (control and test band) indicates the sample is positive for respective DENV, and single control band indicates a valid reaction with negative sample]; Blank normalized (Norm.) black pixel value from the lateral flow strip and corresponding result (+/-); Number of mosquitoes that tested positive (Pos.) compared to the total number (No.) of mosquitoes tested with the respective DENV assay; and Sensitivity of the RT-RPA/LFD assay with 95% confidence interval (CI). *One representative lateral flow strip shown for uninfected mosquito samples.

To apply our DENV 1–4 RT-RPA/LFD assays for mosquito surveillance in low resource settings, we developed a novel method to quickly process mosquitoes for subsequent molecular testing in a format that did not require sophisticated laboratory-based instruments. Our method required only a novel liquid Sample Preparation Reagent, as well as a pestle and microfuge tube for homogenizing the mosquitoes. After 10 minutes incubation on ice, the homogenate was diluted, before 1 µL was used for downstream testing. The combined steps of sample preparation, sample dilution, RT-RPA, and LFD, formed the final version of our rapid DENV 1–4 serotyping tests. We trialled these rapid tests for the detection of DENV-infected and uninfected *Aedes aegypti* mosquitoes ( Figure 5; underlying data ^42^). DENV 1–3 RT-RPA/LFD tests showed 100% congruence with qRT-PCR testing of individual infected (n=8–10) and uninfected mosquitoes (n=5) with 100% diagnostic sensitivity (95% CI= 69% to 100%), as well as 100% diagnostic specificity (CI: 48% to 100%) for respective DENV detection. All except one of the infected mosquitoes that tested positive with qRT-PCR was not detected with DENV 4 RT-RPA/LFD test (11 out of 12 mosquitoes). These results indicated our rapid DENV-4 test had 92% (62% to 100%) diagnostic sensitivity and 100% (CI: 48–100%) diagnostic specificity for respective DENV detection.

**Figure 6.**
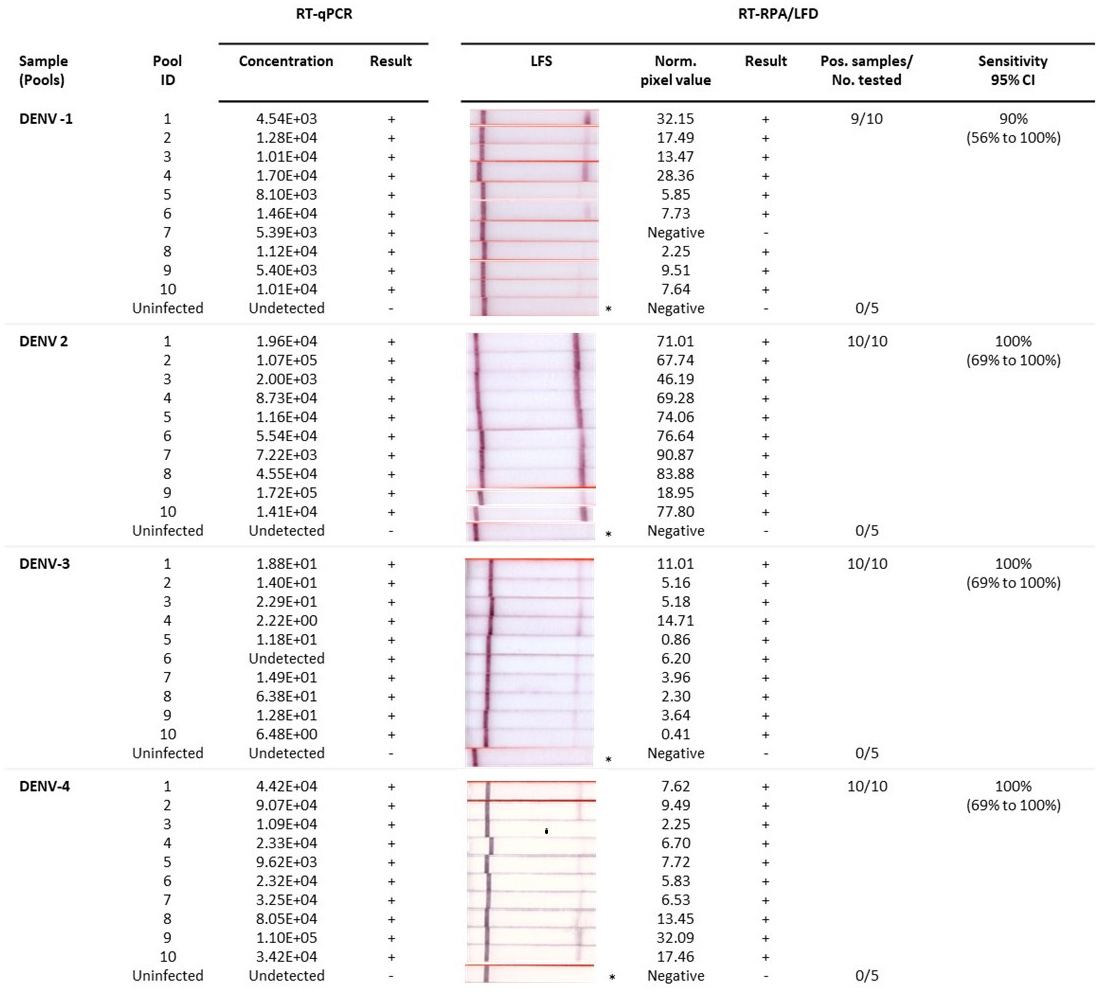
Detection of uninfected and infected mosquito pools by rapid dengue virus (DENV) 1–4 reverse-transcription recombinase polymerase amplification lateral flow detection (RT-RPA/LFD) assays and reverse-transcription quantitative polymerase chain reaction (RT-qPCR). Sample: Pools containing 1 infected DENV-1, DENV-2, DENV-3, or DENV-4 in a pool of 4 uninfected mosquitoes; Pool ID: Serotype-specific mosquito pool identification (ID) number; RT-qPCR: concentration (copies/µL) of respective pooled mosquito head extracts tested and corresponding result (+/-); RT-RPA/LFD: Image of lateral flow strip (LFS) result from RT-RPA/LFD testing the corresponding pooled mosquito body extracts using the respective serotype RT-RPA/LFD test [Images of lateral flow strips with two bands (control and test band) indicates the sample is positive for respective DENV, and single control band indicates a valid reaction with negative sample]; Blank normalized (Norm.) black pixel value from the lateral flow strip and corresponding result (+/-); Number of mosquito pools that tested positive (Pos.) compared to the total number (No.) of mosquito pools tested with the respective DENV assay; and Sensitivity of the RT-RPA/LFD assay with 95% confidence interval (CI). *One representative lateral flow strip shown for uninfected mosquito pool samples.

Following the analysis of individual mosquitoes, we also trialled the detection of DENV 1–4 in pools of mosquitoes containing one DENV infected and four uninfected *Aedes aegypti* mosquitoes. We used the respective virus-specific RT-RPA/LFD for testing the pooled mosquito bodies and the universal DENV 1–4 RT-qPCR for testing the pooled mosquito heads as described above ( Figure 6; underlying data ^42^). Our pooled mosquito testing results indicated that DENV 2–4 RT-RPA/LFD tests showed 100% congruence with qRT-PCR testing with 100% diagnostic sensitivity (95% CI= 69% to 100%), as well as 100% diagnostic specificity (CI: 48% to 100%) for respective DENV detection. Whereas DENV-1 RT-RPA/LFD detected all but one of the infected mosquito pools that tested positive with qRT-PCR (nine out of 10). These results demonstrate 90% diagnostic sensitivity (95% CI= 55.50% to 99.75%) for our rapid DENV-1 test compared to RT-qPCR, as well as 100% diagnostic specificity.

## Discussion

Early detection and identification of DENVs in mosquito populations help determine the infection rate in a mosquito population, indicate the potential for disease outbreaks, and inform control strategies ^1^. While methods such as virus isolation, DENV antigen detection, and RT-qPCR ^43– 46^ are commonly applied for virological surveillance, their inherent infrastructural requirements preclude field implementation, leaving a currently unfulfilled gap for on-site virological surveillance in mosquitoes. In this study, we developed four rapid tests that specifically detect each of the four DENVs in a simple low-resource format, thus facilitating virus detection in mosquito field populations. These tests use a novel rapid sample preparation protocol, a single-temperature isothermal amplification technology (RT-RPA), and simple lateral flow-based detection (LFD) to enable detection of the respective DENV in 35 minutes. These assays have the potential to be used for accurate virological surveillance in mosquitoes without expensive laboratory equipment and the waiting periods involved in sample shipment and testing in a central laboratory. Moreover, the four tests did not detect any closely related flaviviruses presenting with similar clinical symptoms, indicating that the tests are specific to DENV 1–4.

One of the key novelties of our tests is the unique sample preparation method that enables rapid virus detection in crude mosquito extracts suitable for use in a low-resource environment. Preparing samples for nucleic acid testing is a critical step for any field-based mosquito surveillance method, particularly for molecular tests that traditionally require high purity of samples. Previously reported molecular tests employed several mosquito pre-treatment steps such as manual grinding using pestles ^14, 15, 20, 23, 47^, lab-scale milling homogenizers ^16, 18^ and sample disruptors ^21^ followed by the use of expensive commercial RNA extraction reagents and kits, with their time-consuming procedures. These kits restrict sample preparation to labour-intensive, laboratory-based testing, where access to robots, centrifuges and/or vacuum manifolds are available. Contrarily, our rapid and simple protocol reduces sample preparation time to only 10 minutes, involving a single step homogenization in a liquid reagent followed by dilution in RNAse-free water. It alleviates the need for multiple extraction steps and further purification of the extracts. In addition to the rapid sample preparation, our test employs RPA, which has been demonstrated field-applicable for DENV detection in clinical samples using a portable fluorometer, and the studies suggested that RPA is efficient, robust and has high new user acceptance ^26, 29^. By combining our sample preparation with RPA and lateral flow, we describe for the first time a complete method for field-based testing of mosquitoes for detection of the four DENVs. Indeed, further work is required to evaluate the performance and operational utility of these tests in field trials in locations where DENVs are circulating. However, we note that even when processing up to 20 individual mosquitoes or pools in parallel with traditional RNA extraction and RT-PCR, we were able obtain to obtain our rapid test results for all 20 samples before a traditional column-based RNA extraction was completed.

In our study, we used an nfo probe for lateral flow-based detection and distinction between all four DENV serotypes. Nfo probes are intended for detection based on sandwich assays on lateral flow strips, and comprise of a 5’ antigenic label (fluorescein), an internal abasic site (dSpacer or tetrahydrofuran residue) and a 3’ polymerase extension blocking group (C3 Spacer) ^48^. The double strand specific endonuclease IV (nfo enzyme) recognizes and cleaves at the abasic site of the nfo probe only once it is bound to the complementary DNA to form a stable DNA duplex. The cleavage results in generation of a free 3’ OH end and the incised probe then acts as a priming site for the extension by the DNA polymerase. Thus, the nfo probe provides additional specificity to the RPA reaction by adding a critical proofreading step and reduces primer-dependent artifacts ^49^. The majority of previous RPA-based studies have used real-time RT-RPA for detection of all four DENV serotypes with an exo probe testing human samples, but did not distinguish between DENV 1–4 ^26, 27^. One of the reported assays had a detection threshold of about 10–100 copies of DENV RNA from infected cell culture supernatants, with clinical sensitivity and specificity of 77.0% and 97.9% (n=203) in serum samples, respectively ^27^. Another study reported two separate RT-RPA assays for the detection of DENV 1–3 and DENV-4 in plasma, with clinical sensitivity and specificity of 98% (n=31) and 100% (n=23), respectively ^26^. The only other study that used RT-RPA in combination with LFD using nfo-probe developed two different assays to detect DENV 1–3 and 4 separately in clinical samples (n=120) with a 100% coincidence rate with RT-PCR ^31^. Our rapid RT-RPA/LFD dengue tests were 100% accurate compared to RT-PCR for both individual and mosquito pools containing at least 10 ^3^ copies/µL. We do note that analytical sensitivity determined from plasmids in 15 minutes was tenfold better than detection of RNA transcripts in 20 minutes, suggesting a lower relative efficiency of the mMLV reverse transcriptase generating cDNA from an RNA template. This reduction in sensitivity did not appear to affect detection, although DENV-4 was not detected in a single individual mosquito and DENV-1 was not detected in a single pool containing an infected mosquito. Variation in DENV-4 individual mosquito testing could be due to experimental error, whereas the false negative in the DENV-1 pool may be attributed to low viral concentration.

Vector-based identification of individual DENV serotypes co-circulating in an area is vital since all DENV serotypes can cause dengue and severe dengue (SD), and evidence indicates an association between specific DENVs and severity of the disease in subsequent DENV infection ^50^. However, the previously reported RT-RPA tests do not differentiate between individual DENVs, which is a crucial factor in vector-based surveillance. The versatility of the virus-specific tests reported in this study can help identify the potential for outbreaks of the different DENVs. The four dengue viruses share about 65% genome similarity ^51^. Consequently, test specificity is crucial to identify and distinguish between DENVs, as well as other closely related viruses. DENV 1–4 RT-RPA/LFD tests demonstrated 100% analytical specificity (95% CI: 48% to 100%) with respective serotype-specific RNA at 10 ^5^ copies/µL of RNA transcripts.

## Conclusion

In conclusion, we developed rapid tests for detecting each of the four DENVs and demonstrated their suitability for surveillance of viruses in mosquito populations in limited resource settings. We determined the analytical sensitivities of each DENV 1–4 RT-RPA/LFD test to be at least 1000 copies/µL and confirmed all four tests only detected the DENV they were designed to detect and not any other DENV or related flaviviruses. DENV 1–4 RT-RPA/LFD tests also demonstrated excellent diagnostic sensitivity and specificity when applied to individual and pooled infected *Ae. aegypti* mosquitoes compared to a universal dengue RT-qPCR. DENV 1–4 RT-RPA/LFD tests have several advantages compared with conventional methods such as RT-PCR, such as procedural simplicity, rapid sample processing and turnaround time (40 minutes from extraction to detection) and minimal equipment that demonstrates the potential of these tests for field implementation in virus surveillance in mosquito vectors. Since the emergence and spread of severe dengue disease can be attributed to the co-circulation of different DENVs, the early detection of each virus can help to identify the potential for a possible outbreak, and implement timely mitigation strategies. Future field-based trials of the rapid DENV serotyping tests in locations where DENVs are circulating can help establish the operational suitability of these tests for field-caught mosquitoes. If proven successful, the rapid DENV 1–4 RT-RPA/LFD tests can provide an accurate, easy and robust alternative testing method for low-resource DENV surveillance.

## Data Availability

Dryad: Underlying data for ‘Rapid molecular assays for the detection of the four dengue viruses in infected mosquitoes’. https://doi.org/10.5061/dryad.kd51c5b7d ^42^ This project contains the following underlying data: Underlying data for Figure 1 – DENV 1–4 DNA sensitivity test Underlying data for Figure 2 – DENV 1–4 RNA sensitivity test Underlying data for Figure 3 – DENV 1–4 specificity test Underlying data for Figure 4 – DENV 1–4 flavivirus (FV) specificity test Underlying data for Figure 5 – DENV 1–4 Individual mosquito testing Underlying data for Figure 6 – DENV 1–4 Pooled mosquito testing Data are available under the terms of the Creative Commons Zero “No rights reserved” data waiver (CC0 1.0 Public domain dedication). NCBI Genome: Dengue virus 1 isolate. Accession number GU131962.1, https://www.ncbi.nlm.nih.gov/nuccore/GU131962.1 NCBI Genome: Dengue virus 1 isolate. Accession number KF973466.1, https://www.ncbi.nlm.nih.gov/nuccore/KF973466.1 NCBI Genome: Dengue virus 2. Accession number NC_001474.2, https://www.ncbi.nlm.nih.gov/nuccore/NC_001474.2 NCBI Genome: Dengue virus 3 isolate. Accession number KF921921.1, https://www.ncbi.nlm.nih.gov/nuccore/KF921921.1 NCBI Genome: Dengue virus 4 isolate. Accession number JF262780.1, https://www.ncbi.nlm.nih.gov/nuccore/JF262780.1 NCBI Genome: Zika virus culture ATCC:VR-84 isolate MR766. Accession number KX830960, https://www.ncbi.nlm.nih.gov/nuccore/KX830960.1 NCBI Genome: Japanese encephalitis virus strain Nakayama. Accession number EF571853.1, https://www.ncbi.nlm.nih.gov/nuccore/EF571853.1 NCBI Genome: Murray Valley encephalitis virus strain MVE-1-51. Accession number AF161266, https://www.ncbi.nlm.nih.gov/nuccore/AF161266 NCBI Gene: West Nile virus isolate NSW2011. Accession number JN887352, https://www.ncbi.nlm.nih.gov/nuccore/JN887352 NCBI Gene: Dengue virus 1 isolate. Accession number JN415499, https://www.ncbi.nlm.nih.gov/nuccore/JN415499 NCBI Gene: Dengue virus 2 isolate. Accession number JN568254, https://www.ncbi.nlm.nih.gov/nuccore/JN568254 NCBI Gene: Dengue virus 3 isolate. Accession number JN575566, https://www.ncbi.nlm.nih.gov/nuccore/JN575566 NCBI Gene: Dengue virus 4 isolate. Accession number JN575585, https://www.ncbi.nlm.nih.gov/nuccore/JN575585
