# Supplementary material for: Rapid molecular assays for the detection of the four dengue viruses in infected mosquitoes
Source: Gates Open Res. 2022 Dec 22;6:81. Originally published 2022 Jul 19. [Version 2] doi: 10.12688/gatesopenres.13534.2 (PMC9816563; doi:10.12688/gatesopenres.13534.2)
Supplement: Peer review history

# Peer review: Reviewer response for version 2

**DOI:** 10.21956/gatesopenres.15574.r32812

**Published:** 2022-12-23

**Authors:** Carlos Enrique Muskus-López

**Affiliations:** 1 Program for the Study and Control of Tropical Diseases—PECET, Faculty of Medicine, University of Antioquia, Medellin, Colombia

Thank you for sending me the revised version of the article. I believe that in my case the  authors  have satisfactorily answered my questions or comments, for which I have no objection to the manuscript being published.

Is the rationale for developing the new method (or application) clearly explained?

Yes

Is the description of the method technically sound?

Yes

Are the conclusions about the method and its performance adequately supported by the findings presented in the article?

Yes

If any results are presented, are all the source data underlying the results available to ensure full reproducibility?

Yes

Are sufficient details provided to allow replication of the method development and its use by others?

Yes

Reviewer Expertise:

Molecular Biology, Molecular Diagnosis

I confirm that I have read this submission and believe that I have an appropriate level of expertise to confirm that it is of an acceptable scientific standard.

# Peer review: Reviewer response for version 1

**DOI:** 10.21956/gatesopenres.14799.r32736

**Published:** 2022-11-21

**Authors:** Carlos Enrique Muskus-López

**Affiliations:** 1 Program for the Study and Control of Tropical Diseases—PECET, Faculty of Medicine, University of Antioquia, Medellin, Colombia

**Manuscript evaluation:**

 This manuscript shows the design and standardization of a molecular method based on isothermal PCR (Recombinase polymerase amplification or RPA) for the detection and identification of each of the 4 dengue serotypes that infect humans. Additionally, this amplification protocol is combined with a rapid RNA extraction protocol, as well as a reaction development system, based on lateral flow.

 The work is interesting in light of having rapid and cheap diagnostic methods for a disease like dengue, which affects a large number of people in tropical and subtropical areas around the world; but also to monitor the circulation of the different dengue serotypes in the vectors associated with its transmission, which would allow epidemiological surveillance and prediction of possible epidemic outbreaks or dengue complications, if more than one serotypes is circulating in a specific area. Finally, the other interesting thing is the ease of implementing this molecular method of dengue detection in endemic areas with little availability of resources.

 Despite the interestingness of the work, some doubts or comments arise after reviewing the manuscript and which I mention here immediately:

1. In the introduction, second paragraph, it is mentioned that the methods for the surveillance of dengue in mosquitoes include, virus isolation ^10–12^, antigen detection ^13^, and molecular testing using either conventional ^14–17^ or reverse-transcription quantitative polymerase chain reaction (RT-qPCR ) ^18–20^ or isothermal amplification. However, it is mentioned that these methods (including isothermal amplification) are limited by the need for sophisticated equipment and expensive infrastructure, however, the authors contradict themselves later in the text (introduction, Paragraph 3) when they mention that RPA, which is an isothermal amplification method, does not require sophisticated equipment (ref 25), which is true.
2. The authors mention that at least five different forward primers, three reverse primers, and two probes were designed for each of the DENV assays and cite table 1. However, table 1 shows only the selected primers and probe combinations out of the several primers and probes initially designed and tested.
3. It is not clear, why mosquitoes exposed to DENV-2 were infected via feeding on infectious blood meal containing the virus while the rest of the DENV virus were infected via intrathoracic microinjections?
4. What do you think is the reason why the analytical sensitivity determined from plasmids is much better than from RNA? And if this could have implications for using this method to detect dengue virus in humans, where the starting material is just RNA?
5. In the title of Figures 2 and 3 (the text in bold), it is mentioned that the RT-RPA/LFD was run using reverse-transcribed RNA. However, reverse-transcribed RNA literally is cDNA and if I understood well, your starting material in the RT-RPA is just in vitro transcribed RNA from the plasmid, which is different to reverse-transcribed RNA. Of course, during the RT-RPA the RNA is converted in cDNA, just before the amplification.
6. In addition, in the legend of this figure 2, synthetic RNA is mentioned. What do you exactly mean with synthetic RNA? The RNA obtained by in vitro transcription? Because nothing is mentioned about synthetic RNA in materials and methods.
7. In the discussion, it is annotated, “that contrarily, our rapid and simple protocol reduces sample preparation time to only 10 minutes”. This is for preparing how many samples? Maybe 10 minutes might result relative, because if many samples are going to be evaluated (for example in a dengue surveillance study in mosquitoes), this could be time consuming, because of the considerable number of samples to evaluate.

**Minor suggestions:**

1. In Materials and methods; in the plasmid section, the fragment size for DENV-3 (KF921921.1) is missing. In contrast, for the other DENV viruses, the size of the segments is indicated in parentheses.

Is the rationale for developing the new method (or application) clearly explained?

Yes

Is the description of the method technically sound?

Yes

Are the conclusions about the method and its performance adequately supported by the findings presented in the article?

Yes

If any results are presented, are all the source data underlying the results available to ensure full reproducibility?

Yes

Are sufficient details provided to allow replication of the method development and its use by others?

Yes

Reviewer Expertise:

Molecular Biology, Molecular Diagnosis

I confirm that I have read this submission and believe that I have an appropriate level of expertise to confirm that it is of an acceptable scientific standard.

# Response

**Published:** 2022-12-06

**Authors:** Joanne Macdonald

**Affiliations:** University of the Sunshine Coast, Australia

The authors would like to thank the reviewer for their thorough reading of our manuscript and insightful comments. Our responses are below. 

**1.** In the introduction, second paragraph, it is mentioned that the methods for the surveillance of dengue in mosquitoes include, virus isolation10–12, antigen detection13, and molecular testing using either conventional14–17 or reverse-transcription quantitative polymerase chain reaction (RT-qPCR )18–20 or isothermal amplification. However, it is mentioned that these methods (including isothermal amplification) are limited by the need for sophisticated equipment and expensive infrastructure, however, the authors contradict themselves later in the text (introduction, Paragraph 3) when they mention that RPA, which is an isothermal amplification method, does not require sophisticated equipment (ref 25), which is true.

**Response: **We thank the reviewer for their detailed review of our manuscript. The text in paragraph 1 has now been edited to: 

*“ **Apart from isothermal amplification**, these techniques ****can be limited in their implementation due to issues with sensitivity or specificity, or because they require sophisticated instruments and laboratory infrastructure that is not readily available in resource-limited countries where DENVs exert their greatest toll.”*

**2.** The authors mention that at least five different forward primers, three reverse primers, and two probes were designed for each of the DENV assays and cite table 1. However, table 1 shows only the selected primers and probe combinations out of the several primers and probes initially designed and tested.

**Response:** We agree that the citation suggests the table would contain the sequence of all primers and probes. We have now changed the position of the citation to suggest that the table contains only the finalised combinations:

*“At least five different forward primers, three reverse primers, and two probes were designed for each of the DENV assays and tested in various combinations using standard plasmid DNA and synthetic RNA transcripts to optimize the respective serotype specific DENV RPA-LFD assays **, which use selected primer and probe sequences (Table 1).”***

*Table 1. **Selected p**rimer and probe sequences for the Dengue 1-4 RT-RPA/LFD test.*

**3. **It is not clear, why mosquitoes exposed to DENV-2 were infected via feeding on infectious blood meal containing the virus while the rest of the DENV virus were infected via intrathoracic microinjections?

**Response: **To clarify why 2 different methods were used to expose mosquitoes to the viruses, we modified the sentence “ *Mosquitoes were exposed to DENV-2 via feeding on an infectious blood meal containing 10 ^7^ TCID _50_/mL of virus.*” To read as “ *Mosquitoes were exposed to DENV-2 via feeding on an infectious blood meal containing 10 ^7^ TCID _50_/mL of virus, **as part of previously reported experiments (Hall-Mendelin et al. 2021)***”. The reference has been added to the reference list.

**4.** What do you think is the reason why the analytical sensitivity determined from plasmids is much better than from RNA? And if this could have implications for using this method to detect dengue virus in humans, where the starting material is just RNA?

**Response:** We thank the reviewer for the opportunity to further discuss the results of RNA detection compared to plasmid detection. This question has now been addressed in the discussion.

*“Our rapid RT-RPA/LFD dengue tests were 100% accurate compared to RT-PCR for both individual and mosquito pools containing at least 10 ^3^ copies/µL. **We do note that analytical sensitivity determined from plasmids in 15 minutes was tenfold better than detection of RNA transcripts in 20 minutes, suggesting a lower relative efficiency of the mMLV reverse transcriptase generating cDNA from an RNA template. This reduction in sensitivity did not appear to affect detection, although** DENV-4 was not detected in a single individual mosquito and DENV-1 was not detected in a single pool containing an infected mosquito. Variation in DENV-4 individual mosquito testing could be due to experimental error, whereas the false negative in the DENV-1 pool may be attributed to low viral concentration.”*

**5. **In the title of Figures 2 and 3 (the text in bold), it is mentioned that the RT-RPA/LFD was run using reverse-transcribed RNA. However, reverse-transcribed RNA literally is cDNA and if I understood well, your starting material in the RT-RPA is just in vitro transcribed RNA from the plasmid, which is different to reverse-transcribed RNA. Of course, during the RT-RPA the RNA is converted in cDNA, just before the amplification.

**Response:** We thank the reviewer for this comment. We have now replaced the term ‘reverse’ transcribed RNA’ with ‘in vitro’ transcribed RNA in both the figure legends.

**6.** In addition, in the legend of this figure 2, synthetic RNA is mentioned. What do you exactly mean with synthetic RNA? The RNA obtained by in vitro transcription? Because nothing is mentioned about synthetic RNA in materials and methods.

**Response:** Yes, the term synthetic RNA was used interchangeably for the RNA obtained by in vitro transcription. The term has now been excluded and replaced with in-vitro transcribed RNA as described above to avoid confusion.

**7.** In the discussion, it is annotated, “that contrarily, our rapid and simple protocol reduces sample preparation time to only 10 minutes”. This is for preparing how many samples? Maybe 10 minutes might result relative, because if many samples are going to be evaluated (for example in a dengue surveillance study in mosquitoes), this could be time consuming, because of the considerable number of samples to evaluate.

**Response:** We appreciate the reviewer for pointing this out. We have addressed the timing of larger sample sizes in the discussion.

 “ *By combining our sample preparation with RPA and lateral flow, we describe for the first time a complete method for field-based testing of mosquitoes for detection of the four DENVs. Further work is required to evaluate the performance and operational utility of these tests in field trials in locations where DENVs are circulating. **However, we note that even when processing up to 20 individual mosquitoes or pools in parallel with traditional RNA extraction and RT-PCR, we were able to obtain our rapid test results for all 20 samples before a traditional column-based RNA extraction was completed.”***

**Minor suggestions:**

**1.** In Materials and methods; in the plasmid section, the fragment size for DENV-3 (KF921921.1) is missing. In contrast, for the other DENV viruses, the size of the segments is indicated in parentheses.

**Response:** We appreciate the reviewer for pointing this out. The fragment size has now been added in parentheses.

# Peer review: Reviewer response for version 1

**DOI:** 10.21956/gatesopenres.14799.r32271

**Published:** 2022-08-15

**Authors:** Prasad N. Paradkar

**Affiliations:** 1 Australian Centre for Disease Preparedness, CSIRO Health & Biosecurity, Geelong, Vic, Australia

The article by Ahmed *et al. *provides a novel rapid molecular assay based on RT-RPA for detecting DENV in infected mosquitoes. There is a need to effectively monitor the circulation of the dengue virus in low-resource regions. 

 TRT-RPA has previously been used to detect dengue virus from samples. Here the authors have used a simplified and rapid sample processing method, RPA which distinguishes between the serotypes and lateral flow device for easy detection/visualisation. The results showed very high sensitivity and specificity of DENV detection from mosquitoes. Although the number of mosquitoes used for detection was low (8-10 mosquitoes), this provides a pilot testing platform that can be scaled for further validation. This method should also be tested in the field (or using filed samples) for testing suitability.

 The methods are described in detail with sufficient information provided for reproducibility.

 The manuscript offers a suitable method for improving mosquito dengue surveillance efforts in low-resource regions.

Is the rationale for developing the new method (or application) clearly explained?

Yes

Is the description of the method technically sound?

Yes

Are the conclusions about the method and its performance adequately supported by the findings presented in the article?

Yes

If any results are presented, are all the source data underlying the results available to ensure full reproducibility?

Yes

Are sufficient details provided to allow replication of the method development and its use by others?

Yes

Reviewer Expertise:

mosquito-borne diseases, dengue, surveillance, vector-borne disease transmission

I confirm that I have read this submission and believe that I have an appropriate level of expertise to confirm that it is of an acceptable scientific standard.

# Response

**Published:** 2022-12-06

**Authors:** Joanne Macdonald

**Affiliations:** University of the Sunshine Coast, Australia

The authors would like to thank the reviewer very much for their careful reading of the manuscript and encouraging comments.
